# Positive Correlation Between Economic Activities and Fish Diversity in Small River Basins of Less Developed Regions: A Case Study of the Lixian River Basin

**DOI:** 10.3390/ani15162416

**Published:** 2025-08-18

**Authors:** Rong Huang, Bolin Chen, Chengcheng Ma, Chao Deng, Jiaqi Zhang, Zhihui Xiao, Zhijian Wang, Yaqiu Liu, Xiaohong Liu

**Affiliations:** 1 Key Laboratory of Freshwater Fish Reproduction and Development (Ministry of Education), Key Laboratory of Aquatic Science of Chongqing, School of Life Sciences, Southwest University, Chongqing 400715, China; ronghuang0923@163.com (R.H.); 15123315281@139.com (B.C.); a1729522996@163.com (C.M.); dc17749941501@163.com (C.D.); 15086991418@163.com (J.Z.); 15023909974@163.com (Z.X.); wangzj1969@126.com (Z.W.); 2 School of Agronomy, Xinjiang Hetian College, Xinjiang 848000, China; 3 Pearl River Fisheries Research Institute, Chinese Academy of Fishery Sciences, Guangzhou 510380, China

**Keywords:** fish diversity, environmental DNA, economic development

## Abstract

(1) Background: Affected by multiple factors, the decline in fish species diversity in some aquatic ecosystems has become increasingly pronounced. At a broad spatial scale, economic development has been widely recognized as one of the key factors influencing the fish distribution pattern. However, at a small scale, within a single river basin, the effects of economic development on the freshwater fish distribution and communities remain largely uninvestigated. (2) Methods: environmental DNA (eDNA) samples were collected from 26 sampling sites of the Lixian River in both the summer (June) and winter (November). Economic data from the Lixian River basin were collected, and analyses, including multivariate regression tree analysis and generalized linear model fitting, were performed using R 4.3.2. (3) Results: A total of 65 fish species was characterized, and the Chao1 diversity indices in the upstream (13.42) and downstream (13.00) were significantly higher than those in the middle reaches (8.55, *p* < 0.01) of this river. The species communities exhibited an obvious gradient changing pattern from the upstream to the downstream reaches, with parameters of water quality, including transparency, pH, dissolved oxygen and temperature, and climatic factors functioning as the key variables. Furthermore, the generalized linear model analysis revealed significant positive correlations between agricultural population (*p* = 0.00106), total grain production (*p* = 0.00476), total population (*p* = 0.00192) and the Chao1 index. (4) Conclusions: Climatic factors are the key factors affecting the fish diversity in the Lixian River. In less economically developed areas, the development of local economic activities may enhance fish diversity.

## Simple Summary

Economic activities have a significant impact on the aquatic environment, including fish. In this study, we employed environmental DNA (eDNA) technology to conduct surveys in the Lixian River basin, where economic development exhibits a gradient pattern. The results indicated that fish communities showed an obvious gradient variation from the upstream to the downstream reaches. Surprisingly, we found significant positive correlations between agricultural population, total grain production, total population, and the diversity index. This suggests that in less economically developed regions, the development of local economic activities may enhance fish diversity, revealing a potential synergistic relationship between economic activities and fish diversity in small-scale river basins and providing a new perspective for understanding economic–ecological interactions.

## 1. Introduction

Over 10,000 fish species inhabit freshwater environments around the world [1]. They account for about 40% of global fish and one-quarter of all vertebrate species [2]. Over the past half century, freshwater ecosystems have experienced a significant decline in fish species diversity due to habitat destruction, climate change, the introduction of non-native species, and so on [3,4]. Changes in fish species composition and distribution always serve as effective indicators of aquatic ecosystems health; thus, monitoring fish diversity has long been proposed as a critical first step in developing and implementing effective conservation and management strategies [5].

Currently, there is no consensus on the main drivers shaping fish diversity and community composition [6]. Natural biogeographic factors such as climate and geographic features are widely recognized as interactively affecting key factors in large-scale fish diversity and distribution [7,8,9]. However, in recent years, human-induced disturbances have significantly altered environmental conditions and profoundly impacted biodiversity patterns [10]. In addition, socio-economic factors, including population growth, economic development, urbanization, and fishery-related activities such as overfishing and the introduction of non-native species, have increasingly emerged as significant drivers of fish diversity [4,11,12]. Therefore, quantifying the effects of anthropogenic factors on biodiversity is crucial for ensuring the health and sustainable development of ecosystems [13]. Existing research on large-scale freshwater systems has consistently shown that socio-economic development is a key factor contributing to the decline in fish diversity. Specifically, the population and economic growth in North America has been reported to be the primary drivers of freshwater fish diversity loss [14]. Similarly, the deleterious effects of rising national economic level on threatened fish species have been pointed out earlier [15]. In addition, a study found that the species richness of freshwater fish is not only related to physical geography and climate factors such as rainfall, air temperature, and the area of surface water bodies, but also positively correlated with the inland fishery production in China [16]. However, at a small scale, within a single river basin, the effects of economic development on the freshwater communities remain largely uninvestigated.

The environmental DNA (eDNA) technique has emerged as an innovative and effective technology in fish diversity monitoring in recent years [17]. Compared to traditional fishing methods, the eDNA technique offers several advantages, including high sensitivity and efficiency, cost-effectiveness, and environmental friendliness [18]. Besides fish diversity, this technique has also been extensively applied to the monitoring of invasive aquatic species [19,20], the tracking of endangered species [21,22], and the assessment of species abundance and biomass across various aquatic organisms [23,24]. Notably, this technique can successfully detect target species at densities as low as 1–2 individuals per square kilometer [25]. Additionally, by integrating environmental factor data, the eDNA technique can effectively address challenges that traditional methods cannot overcome. For example, by employing an eDNA-based multitrophic-level biological monitoring dataset, the interactive effects of dams and nutrient enrichment on aquatic communities at the levels of α-diversity, β-diversity, and food webs have been successfully demonstrated [26].

The aim of this work was to investigate the influence of economic development on the diversity and distribution patterns of freshwater fish within a relatively small-scale area, to understand the composition and distribution of fish in the Lixian River of Yunnan, and to provide new insights into the ecological conservation of mountainous river ecosystems.

## 2. Materials and Methods

### 2.1. Research Area

**Figure 1:**
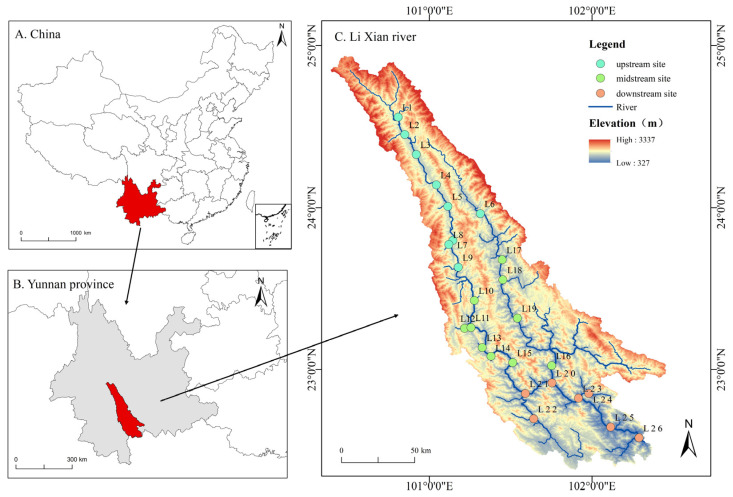
Distribution of eDNA water sampling sites in the Lixian River. (**A**) Map of China, with Yunnan Province in red. (**B**) Map of Yunnan Province, with Lixian River basin in red. (**C**) Map of the Lixian River basin, where L1–L26 indicates the sampling sites, with fill colors of blue, green, and orange indicating the upstream, midstream, and downstream sites, respectively.

The Lixian River, a first-order tributary of the Honghe River, stretches for 427 km within Yunnan Province (Figure 1). As a typical mountainous river, it is characterized by fast water flow and rich fish resources [27,28]. Originating in Dali, this river flows through several counties with different levels of socio-economic development, including Jingdong Yi Autonomous County, Zhenyuan Yi, Hani and Lahu Autonomous County, Mojiang Hani Autonomous County, Ning’er Hani and Yi Autonomous County, and Jiangcheng Hani and Yi Autonomous County.

### 2.2. Sample Collection

A total of 26 sampling sites were set up within the Lixian River basin (Figure 1). L1–L9, L10–L19, and L20–L26 were located in the upstream, midstream, and downstream reaches of this river, respectively. Following the protocol of a previous study [29], environmental DNA (eDNA) samples were collected during both summer (June) and winter (November) of 2023 using a portable eDNA sampler (Model WY-103, Shanghai Weiyu Technology, Shanghai, China). In brief, three replicates and one negative control were collected at each site in each season, and all samples were filtered using a 0.45 μm mixed cellulose ester membrane under vacuum within 24 h. After the filtration, all membranes were stored at −20 °C until use.

During the sample collection at each site, indicators of water quality, including dissolved oxygen (DO), water temperature (Temp), pH, electrical conductivity (EC), salinity (SAL), and water transparency (WT), were simultaneously measured by a portable dissolved oxygen tester (HACH, Loveland, CO, USA), a pH meter, a conductivity meter, a salinity meter (all SMART SENSOR, Shanghai, China), and a Secchi disk (Table S1).

### 2.3. Total eDNA Extraction and Sequencing

eDNA was extracted from the filtered membranes using the Fast DNA^®^ SPIN Kit for Soil (MP Biomedicals, Irvine, CA, USA), following the manufacturer’s protocol. The integrity and quality of each extracted DNA were evaluated by agarose gel electrophoresis, and quantification was performed using a NanoDrop 2000 spectrophotometer (Thermo Fisher Scientific, Waltham, MA, USA) and a Qubit 3.0 fluorometer (Thermo Fisher Scientific, Waltham, MA, USA). The mitochondrial 12S rRNA gene hypervariable region was amplified using Tele02_F/R (Tele02-F: 5′-AAACTCGTGCCAGCCACC-3′; Tele02-R: 5′-GGGTATCTAATCCCAGTTTG-3′ [30]). The PCR was carried out in a 10 μL volume, consisting of 1 μL 10× Toptaq buffer (Transgen, Beijing, China), 0.8 μL dNTPs (2.5 mM), 2 μL template DNA, 0.2 μL each of forward and reverse primers (10 μM), and 0.2 μL Toptaq DNA Polymerase (Transgen, Beijing, China). The thermal conditions consisted of an initial denaturation at 95 °C for 2 min, followed by 30 cycles of 95 °C (30 s), 55 °C (30 s), and 72 °C (1 min), and a final extension at 72 °C for 10 min. Each sample was amplified in triplicate, including negative controls (no template) and filter blanks. PCR products were purified using Agencourt AMPure XP Beads (Beckman Coulter, Brea, CA, USA), and then followed by indexed libraries construction, repurification, quantification, normalization, and finally sequencing on an Illumina NovaSeq 6000 platform (Illumina, San Diego, CA, USA) using a paired-end 250 bp (PE250) SP-Xp strategy.

### 2.4. Economic Data Collection of Yunnan’s Counties and Districts in the Lixian River Basin

A total of 19 economic indicators from 2013 to 2022 were selected to characterize the level of overall economic, agricultural, breeding, and industrial development across the upper, middle, and lower reaches of the Lixian River basin (Table S2). Provincial-level data were sourced from the Yunnan Statistical Yearbook (2013–2022), while national-level datasets were obtained from the China Regional Economic Database and China Urban and Rural Construction Database (https://www.epsnet.com.cn). Hydrological data were provided by the Pu’er City Water Conservancy Department, and climatic records were accessed via the Xihe Energy Big Data Platform (https://xihe-energy.com).

### 2.5. Bioinformatic Analyses

After removing of adapter and primer sequences using QIIME2 software [31], the DADA2 plugin [32] was employed for quality control, noise reduction, sequence splicing, and chimera removal. Species accumulation and rarefaction curves were generated to evaluate the sampling rationality (Figure S1). The USEARCH (v.10.0) was used to cluster the sequences into Operational Taxonomic Units (OTUs) at a 97% sequence similarity threshold [33], and a BLAST search with the MitoFish V.4.09 database was also performed. What is more, manual corrections were made based on the historical distribution information of fish in the Lixian River basin, referring to the atlas of Yunnan fishes [34], color atlas of native fishes in Pu’er [35], fauna sinica, and species and distribution of inland fishes in China [36] to ensure the accuracy of species annotation. A fish species was only considered present in a sample when the number of its sequence was equal to or greater than 3 [37].

### 2.6. Statistics

The amplicon sequence variant (ASV) abundances were normalized using Phyloseq V1.2.6 based on a previous report [38]. α-diversity, including the Chao1, the Shannon–Wiener index, Simpson’s diversity, and Pielou’s evenness index were computed using the vegan package V2.6.8 [39] in R.

One-way ANOVA was performed to test differences among different reaches of the Lixian River (significance at *p* < 0.05). A principal co-ordinates analysis (PCoA) based on Bray–Curtis dissimilarity matrices [40] was conducted to investigate spatio-temporal variations in fish community structure, and the permutational multivariate analysis of variance (PERMANOVA) was applied to assess significance. Canonical Correlation Analysis (CCA) was employed to analyze the effects of environmental factors on fish communities.

Using Yunnan Province’s per capita GDP as the benchmark (regions below classified as less economically developed), the six counties mentioned all had significantly lower per capita GDP than the provincial level (Figure S2, one-way ANOVA, *p* < 0.05), confirming the basin’s status as less economically developed. A Pearson correlation analysis was performed on 19 selected economic indicators, and those with strong correlations (r > 0.7, *p* < 0.05) were excluded (Figure S3). Finally, variables including GDP, population, agricultural population, total grain output, aquatic product yield, number of hogs slaughtered and industrial gross output were retained, representing overall economy, agriculture, aquaculture, animal husbandry, and industrial development for subsequent analyses.

A three-step framework explored links between economic development and fish diversity: (1) Independent multiple regression tree models were constructed for the four α-diversity indices to identify the underlying determinants, while 10-fold cross-validation and the one-standard-error (1-SE) rule [11] were used to prune regression trees. (2) Generalized linear models (GLMs) tested economic factors’ effects while controlling for dominant natural drivers. (3) A partial least-squares path modeling (PLS-PM) analysis with 17 key indicators revealed mechanisms: water quality variables (permanganate index, total phosphorus, total nitrogen, pH, water temperature, total ammonia nitrogen, salinity); overall economic variables (per capita GDP, total population); agricultural variables (grain yield, aquatic product yield, hog slaughter numbers); industrial variables (total industrial output); climate variables (minimum/average temperature).

In this study, all data analyses were completed by R4.3.2 [41]. Specifically, the multiple regression tree was carried out using the mvpart package (v1.6-2) [42], and the GLM was performed using the MASS package [43]. The PLS-PM fitting analysis was conducted by plspm (v0.5.1) [44] and semPlot (v1.1.6) [45]. Finally, ggplot2 [46] was used for visualization.

## 3. Results

### 3.1. Species Identification and Composition in the Lixian River

**Figure 2:**
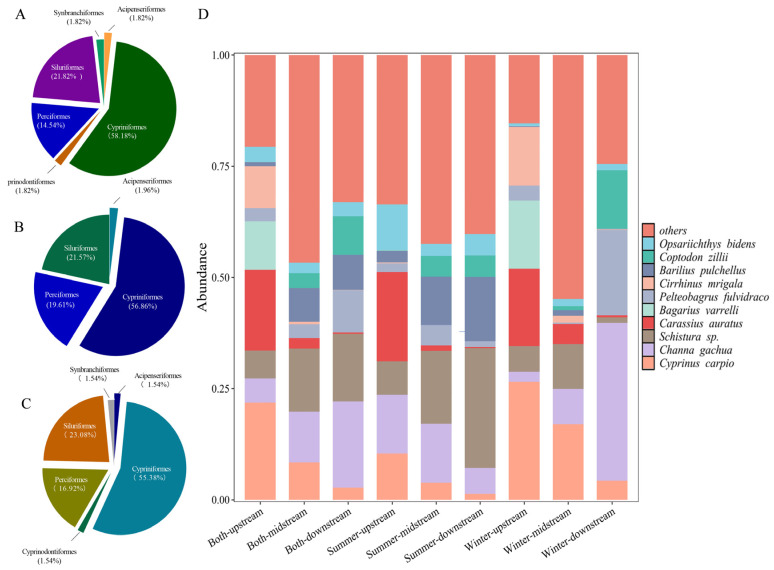
Fish species composition in the Lixian River. (**A**) Fish composition at the order level during the summer; (**B**) fish composition at the order level during the winter; (**C**) fish composition at the order level across both seasons; (**D**) relative sequence abundance of fish species during the summer, winter, and across both seasons in the upstream, midstream, and downstream reaches.

A total of 55 species/genera belonging to 45 genera, 16 families, and 6 orders in summer, and 51 species/genera belonging to 40 genera, 13 families, and 4 orders in winter were identified, respectively. In both seasons, the order Cypriniformes was the most dominant, with species accounting for more than 55% of the total, followed by the Siluriformes (more than 20%), and by the Perciformes (14.55% and 19.61 in summer and winter, respectively). The other three orders, including Acipenseriformes, Synbranchiformes, and Cyprinodontiformes, each comprised only one species (Figure 2A,B).

Combining data from the two seasons, a total of 65 species/genera, belonging to 49 genera, 17 families, and 6 orders were identified. The Cypriniformes remained the most diverse order, with 36 species (55.38%), followed by the Siluriformes, with 15 species (23.08%), and the Perciformes, with 11 species (16.92%). Three orders, Acipenseriformes, Synbranchiformes, and Cyprinodontiformes, each comprised just one species, accounting for 1.54% of the total (Figure 2C). The dominant species were *C. carpio*, *C. gachua,* and *Schistura* sp. (Figure 2D).

### 3.2. Fish Diversity and Spatial Distribution Pattern in the Lixian River

#### 3.2.1. Fish Diversity in the Upstream, Midstream and Downstream Reaches of the Lixian River

**Figure 3:**
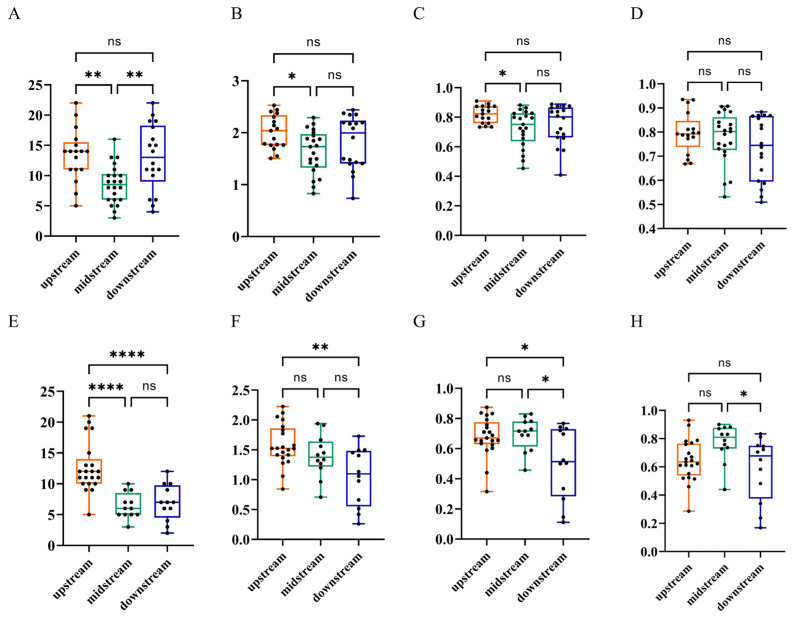
Fish α-diversity in the upstream, midstream, and downstream reaches of the Lixian River. (**A**–**D**) Chao1 index, Shannon–Wiener index, Simpson index, and Pielou index during summer; (**E**–**H**) Chao1 index, Shannon index, Simpson index, Pielou index during winter. ****: *p* < 0.0001, **: *p* < 0.01, *: *p* < 0.05, ns: non-significance.

During summer, significantly higher Chao1 indices were observed in both the upper and lower reaches than in the middle reach (*p* = 0.0031, 0.0062, respectively; Figure 3A), highlighting pronounced longitudinal variations in species richness. Notably, the Simpson and Shannon–Wiener indices of the downstream reach were significantly greater than those of the midstream reach (*p* = 0.0235, 0.0351, respectively; Figure 3B,C). The Pielou index exhibited the highest value in the upstream and the lowest in the downstream reaches, but without reaching significance (Figure 3D). During winter, the Chao1 index of the upstream reach was significantly higher than those of both the midstream and downstream reaches in winter (*p* < 0.0001, Figure 3E), reflecting notably greater species richness in the upstream section. However, the Shannon–Wiener index of the downstream reach was significantly higher than that of the upstream reach (*p* = 0.0015, Figure 3F). The Simpson index in the downstream was significantly lower than those in both the midstream and upstream reaches (*p* = 0.0128 and 0.0154, respectively; Figure 3G), while the Pielou index in the midstream reach was significantly higher than that in the downstream reach (*p* = 0.0272, Figure 3H). These results indicted a spatio-temporal-dependent effects of fish diversity in the Lixian River.

#### 3.2.2. Relationship Between the Spatial Distribution Pattern of Fish and Natural Environmental Factors

**Figure 4:**
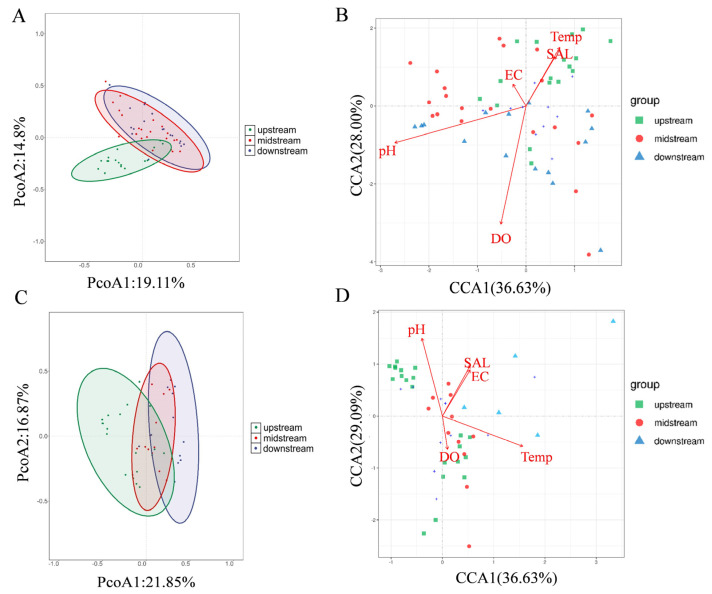
Drivers of fish community spatial patterns and key water quality variables in the Lixian River. (**A**,**B**) PCoA analysis results and CCA analysis results during summer; (**C**,**D**) PCoA analysis results and CCA analysis results during winter.

To verify the reach-dependent effects on fish diversity, the β-diversity was also analyzed in the three reaches of this river. As expected, fish communities differed significantly among the upstream, midstream, and downstream reaches of the Lixian River (*p* < 0.01), forming an obvious gradient from the upstream to downstream reaches (Figure 4A,C). In addition, the effects of water quality indicators on the spatial distribution pattern of fish communities in the Lixian River showed a season-dependent manner. Specifically, the key driving factors in summer were pH (R^2^ = 0.4286, *p* = 0.001) and dissolved oxygen (DO, R^2^ = 0.4075, *p* = 0.001), while in winter the factors shifted to water temperature (Temp, R^2^ = 0.8299, *p* < 0.001) and pH (R^2^ = 0.6029, *p* = 0.009) (Figure 4B,D).

### 3.3. The Impacts of Economic Development Level on the Fish Diversity in the Lixian River

#### 3.3.1. Economic Development and Climate Status of the Upper, Middle, and Lower Reaches of the Lixian River

**Figure 5:**
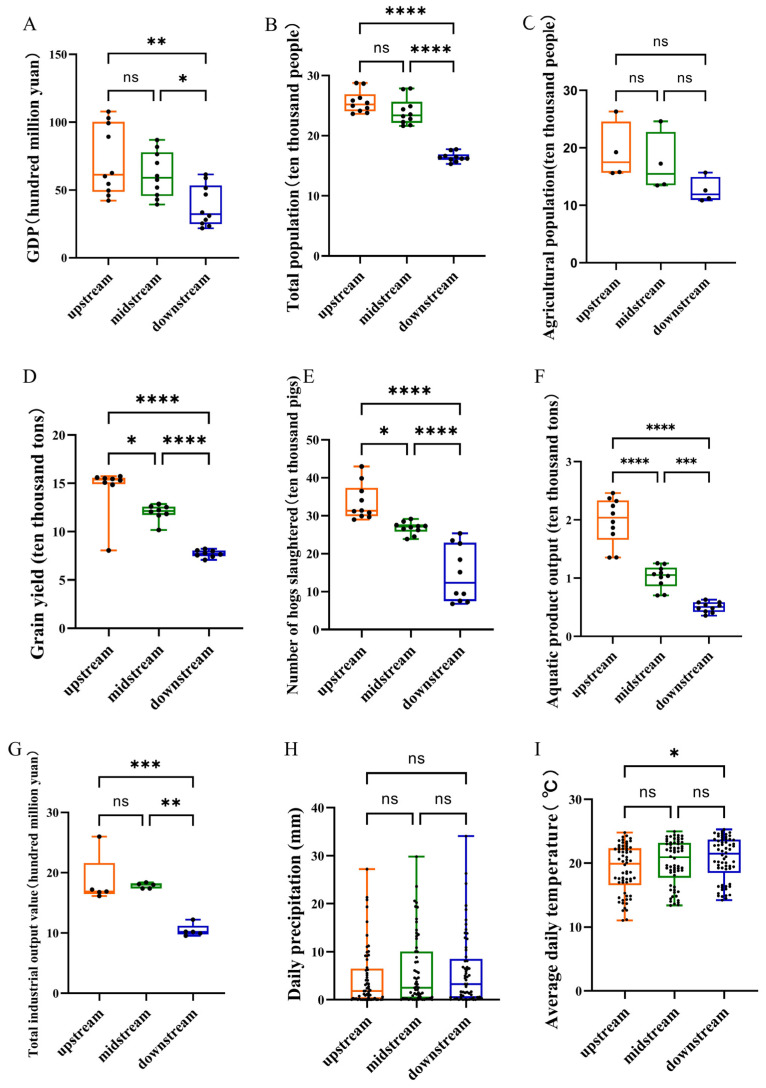
Economic development and climate in the Lixian River. (**A**) GDP; (**B**) total population; (**C**) agricultural population; (**D**) grain yield; (**E**) number of hogs slaughtered; (**F**) aquatic product output; (**G**) total industrial output value; (**H**) daily precipitation; (**I**) average daily temperature. ****: *p* < 0.0001, ***: *p* < 0.001, **: *p* < 0.01, *: *p* < 0.05, ns: non-significance.

The development levels of overall economy, agriculture, and breeding industry in the Lixian River basin presented a gradually decreasing trend from the upstream to the downstream. However, industrial development is more pronounced in the middle reach. Specifically, GDP and total population in both the upstream and midstream reaches were significantly higher than that of the downstream (*p* < 0.05; Figure 5A,B). In the agricultural sector and breeding industry, except for agricultural population, all other indices, including grain output, number of hogs slaughtered, and aquatic product output, exhibited a distinct gradient: the upstream significantly outperformed the midstream, which in turn surpassed the downstream significantly (*p* < 0.05, Figure 5C–F). For the industrial index, the total industrial output value revealed that both the upstream and middle reaches were significantly more developed than the downstream reaches (*p* = 0.0006 and 0.0013, respectively), whereas the difference between the upstream and middle reaches was non-significant (*p* = 0.8802, Figure 5G).

In terms of climatic indices, there was no significant difference in daily precipitation across the basin; however, the downstream region showed a significantly higher daily temperature than the upstream region (*p* = 0.0311, Figure 5H,I).

#### 3.3.2. The Relationship Between Fish Diversity and Economic Development in the Lixian River

**Figure 6:**
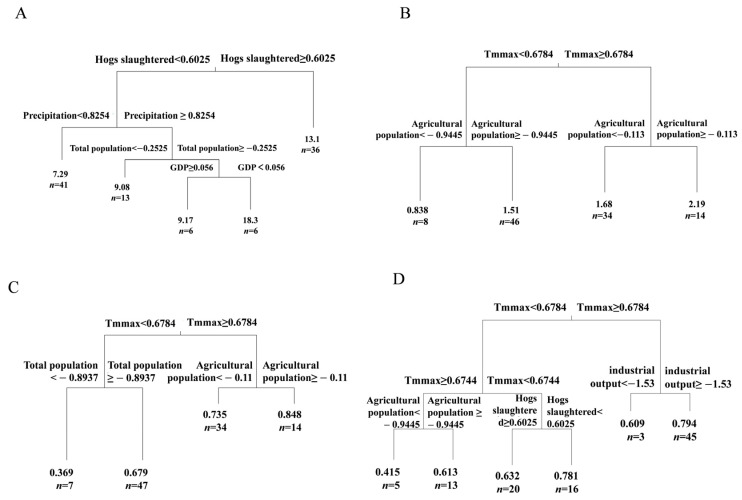
Multiple regression trees based on fish diversity. (**A**) Regression tree analysis based on the Chao1 index; (**B**) regression tree analysis based on the Simpson index; (**C**) regression tree analysis based on the Shannon–Wiener index; (**D**) regression tree analysis based on the Pielou index.

The regression tree analyses were separately conducted for each of the four aforementioned diversity indices (Figure 6). In terms of the Chao1 index, the primary split was defined by the number of hogs slaughtered, followed by precipitation, whereas the Shannon–Wiener index was firstly divided by the maximum temperature (Tmmax) and then by the agricultural population. Similarly, the Simpson index was initially divided by the maximum temperature (Tmmax) and further subdivided by factors such as total population and agricultural population. The primary split of the Pielou index was defined by the maximum temperature (Tmmax), followed by the total industrial output value (industrial output). These results suggested that climatic factors, particularly the maximum temperature, exhibited stronger predictive power compared to human-driven factors. However, the impacts of human-driven factors, such as agriculture and breeding industry, on fish diversity remained significant and nonnegligible.

**Figure 7:**
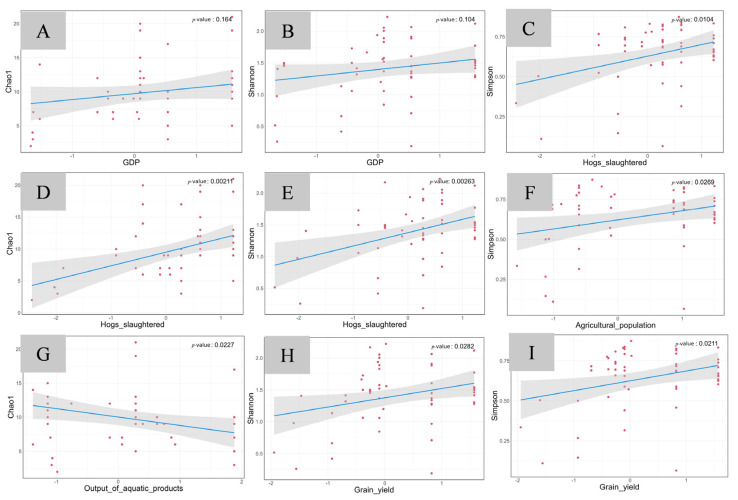
Generalized linear model analysis of fish diversity indices and indicators of economic activities in the group with lower maximum temperature. The horizontal axis represents economic indicator factors, and the vertical axis represents the diversity indices. (**A**) GDP–Chao1; (**B**) GDP–Shannon; (**C**) hogs slaughtered–Simpson; (**D**) hogs slaughtered–Chao1; (**E**) hogs slaughtered–Shannon; (**F**) agricultural population–Simpson; (**G**) output of aquatic product–Chao1; (**H**) grain yield–Shannon; (**I**) grain yield–Simpson.

Within the two subsets, divided based on the maximum temperature (Tmmax) as the key controlling factor, anthropogenic economic factors exhibited similar relationships with fish diversity indices. In the group with lower maximum temperature, the number of hogs slaughtered showed significant positive correlations with all three fish diversity indexes (*p* < 0.05, Figure 7C–E). Grain yield exhibited significant positive correlations with both the Shannon–Wiener (*p* = 0.0282, Figure 7H) and the Simpson indexes (*p* = 0.0211, Figure 7I). Agricultural population only showed a significant positive correlation with the Simpson index (*p* = 0.0269, Figure 7F). GDP showed a positive but insignificant correlation with the Chao1 index (*p* = 0.164, Figure 7A) and the Shannon index (*p* = 0.104, Figure 7B), while aquatic product output showed a negative correlation with the Chao1 index (*p* = 0.0277, Figure 7G).

**Figure 8:**
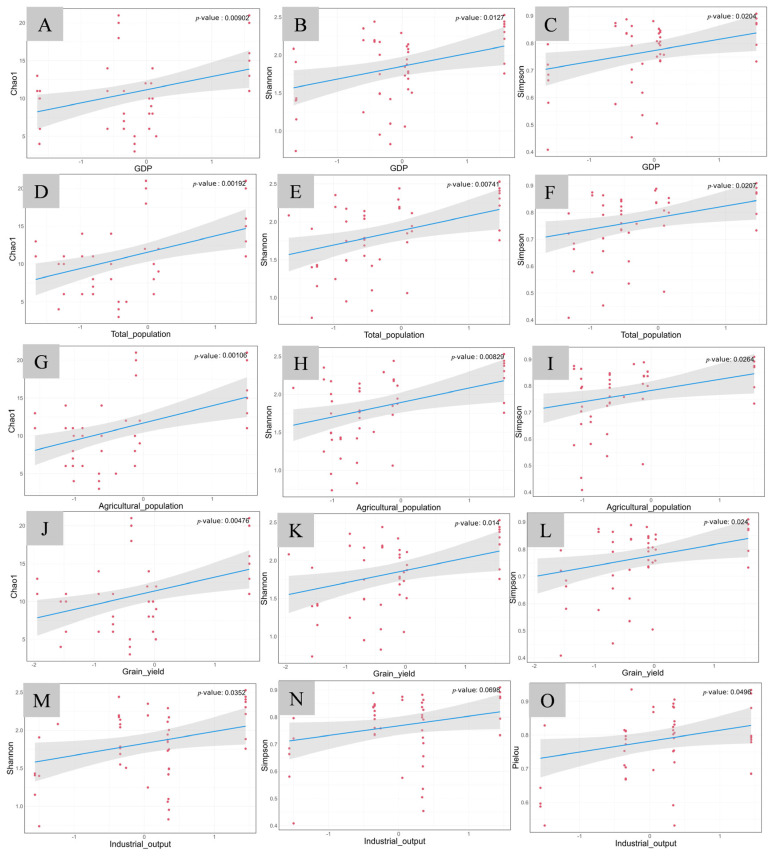
Generalized linear model analysis of fish diversity indices and indicators of economic activities in the group with higher maximum temperature. The horizontal axis represents economic indicator factors, and the vertical axis represents the diversity indices. (**A**) GDP–Chao1; (**B**) GDP–Shannon; (**C**) GDP–Simpson; (**D**) total population–Chao1; (**E**) total population–Shannon; (**F**) total population–Simpson; (**G**) agricultural population–Chao1; (**H**) agricultural population–Shannon; (**I**) agricultural population–Simpson; (**J**) grain yield–Chao1; (**K**) grain yield–Shannon; (**L**) grain yield–Simpson; (**M**) industrial output–Shannon; (**N**) industrial output–Simpson; (**O**) industrial output–Pielou.

In the group with higher maximum temperature, GDP, total population, and agricultural population, grain yield demonstrated significant positive correlations with all three fish diversity indexes (*p* < 0.05, Figure 8A–L), whereas the industrial output showed significant positive correlations with both the Shannon–Wiener index (*p* = 0.0352, Figure 8M) and the Pielou index (*p* = 0.0496, Figure 8O).

Based on the above results, it is obvious that local economic activities, particularly the agricultural and breeding industrial aspects, have positive influences on fish diversity in the Lixian River basin, which exhibits a less economically developed level.

### 3.4. Mechanisms of Effects of Economic Development Level on Fish Diversity in the Lixian River

**Figure 9:**
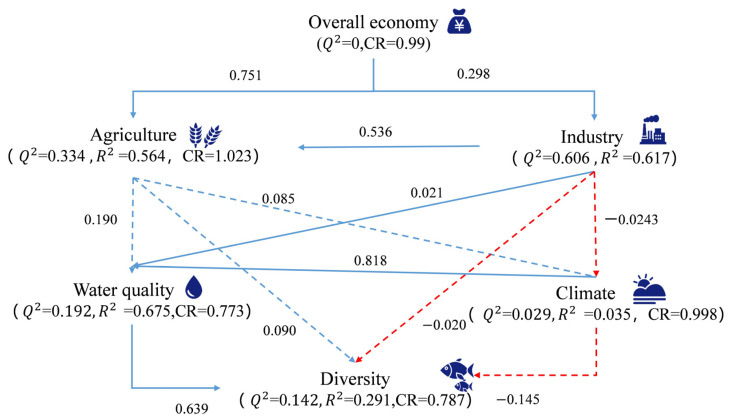
Partial least-squares path model based on the economy, fish diversity, and environment of the Lixian River. Red and blue arrows represent negative and positive impacts, respectively. Solid lines indicate significance (*p* < 0.05), and dashed lines indicate non-significance (*p* > 0.05). The HTMT (Heterotrait–Monotrait ratio) values in the PLS-PM model (Table S3) are all less than 0.9, all Q^2^ values are greater than 0, and all CR (Composite Reliability) values are greater than 0.7, indicating that the model fits well.

To explore the mechanism by which economic factors affect fish diversity in the Lixian river basin, a partial least-squares path model (PLS-PM) was employed (Figure 9). The overall economy showed a direct positive relationship with agriculture and industry (*p* < 0.0001). Agriculture was positively related to fish diversity, while industry showed a negative relationship. Climate exhibited a weak negative impact on fish diversity, while water quality had a significant positive direct effect (*p* = 0.0046), indicating its importance as a critical factor in shaping fish diversity. Agriculture exerted a positive influence on both water quality and climate, as indicated by path coefficients of 0.190 and 0.085, respectively. In contrast, industry showed a tendency toward a positive impact on water quality (path coefficient: 0.021) and a tendency toward a negative impact on climate (path coefficient: −0.0243).

## 4. Discussion

### 4.1. Changes in the Fish Composition of the Lixian River Basin

In the present study, a total of 65 fish were identified by eDNA in the Lixian River, and 29 of which were also historically recorded in data from fish capture in this river approximately 15 year ago [27]. Among these identified fish, it was hard to distinguish *Schistura callichroma* and *Schistura fasciolatus* at the species level, due to the primer discrimination, which resulted in the matching degrees of these two fish being above 97% and a difference between them of less than 1%. When comparing the species composition, a notable increase in the proportion of non-native fish was found in the present study. In 2010, the number of non-native fish individuals accounted for only a small proportion (3%) of all fish catches, while the current relative sequence abundance of non-native fish has risen to 18.28%. In addition, there were three species of tilapia identified in the present study, including *Oreochromis niloticus*, *O.mossambicus*, and *Coptodon zillii*, and all three fish ranked among the top 10 species in terms of relative sequence abundance during the winter (Figure 2). The tilapias are known for their strong adaptability and reproductive capacity [47]. Yunnan Province is one of the main farming regions of tilapia in China [48], and the escape of tilapia from farms to natural rivers is inevitable. Based on the recorded data of water temperature during sample collection, and those retrieved from the official database, the minimum water temperature in winter in the Lixian River is higher than 18 °C, which provides a suitable environment for the escaped tilapia from farming populations, consequently leading to the rapid establishment of a dominant community of these invasive fish in the Lixian River within just over a decade.

### 4.2. Effects of Climate on the Fish Diversity in the Lixian River

In the present study, both CCA and regression tree analyses supported the conclusion that natural factors were important in shaping fish diversity in the Lixian River (Figure 4 and Figure 6). Climate change is the one that received most extensive attention, and it has been widely recognized as a key driver of biological shifts in natural systems [49]. As climate change progresses, the frequency of floods and droughts is increasing, which inevitably leads to a rise in water engineering projects [50]. This, in turn, intensifies alterations in water flow patterns and exerts a profound impact on fish populations [51]. Not just water flow, but climate change-mediated alterations in many other natural factors, including temperature, precipitation, augmented drought occurrences, and early-onset floods, have been reported to result in a decline of 21.25% in fish biodiversity in Bangladesh [52]. Climate change can directly affect the physiological activities of fish as well [53]. For instance, the raised water temperature driven by increased air temperature can elevate fish metabolic rate, thereby resulting in a higher oxygen demand; however, higher water temperature is always accompanied by a decline in dissolved oxygen. These dual effects finally result in an insufficiency of dissolved oxygen, which constrains fish growth, reproductive capabilities, and other physiological activities [54]. In addition, under the challenge of global climate change, alterations in the frequency and intensity of extreme climate events (such as heavy rainfall, droughts, extreme high temperatures, etc.) can also profoundly affect fish reproduction [55], migration [56], and even survival [57]. Moreover, climate-mediated environmental indicators are also closely related to changes in fish distribution. For example, in response to the rising water temperature, the distribution ranges of many fish species along the coastline of Texas have expanded from relatively southern regions towards the north over the past 35 years [58]. Also driven by climate change, the geographic redistribution of marine fish species has been reported, with an increase in species richness in the Arctic within the past 20 years [59]. In Norway, climate warming has led to a poleward shift in fish distribution, changing the fish community from an obvious latitudinal pattern to a more homogeneous one [60]. Similarly, in the Nordic Seas, the raising seawater temperature has caused fish distributions to shift toward more northerly or deeper waters [61]. What is more, the redistribution of invasive fish species has become a highly concerning issue in recent years, and the impacts of invasive fish on local biodiversity will be amplified or exacerbated due to climate warming [62].

### 4.3. The Impact of Economic Development on Fish Diversity in the Lixian River

In this study, although the overall economic and agricultural development levels in the Lixian River basin showed a gradually decreasing pattern from the upstream to the downstream regions, higher fish diversity was observed in both the upstream and downstream areas (Figure 3 and Figure 4), indicating a nonlinear relationship between economic development and fish diversity. The PLS-PM analysis here also indicates a complex relationship between economic levels and fish diversity. This is consistent with the previous view that the relationship between economic development and fish richness and diversity is influenced by multiple complex mechanisms [15].

Contrary to the traditional view that economic development often has a detrimental impact on fish diversity [11,63], the present study found that in regions with relatively low level of economic development, local economic activities, especially those related to agriculture and aquaculture, can positively influence fish diversity (Figure 7 and Figure 8). This was verified by the significant and positive impacts of agriculture on fish diversity in the PLS-PM analysis (Figure 9). Although the degradation of river connectivity by dams or other human activities globally threatens the freshwater biodiversity [5], moderate agricultural activities, such as the rational management of irrigation water, may maintain the hydrological connectivity of rivers, thus providing suitable habitats and migration environments for fish [64,65], and thereby sustaining biodiversity [66]. Freshwater ecosystems encompass not only the humid peripheries but also the catchment areas that serve as sources of water and nutrients [2]. Moreover, some pond aquaculture systems, to a certain extent, simulate the natural wetland ecology, offering diverse habitats and food sources for fish [67]. The available research has shown that low-intensity agriculture, characteristic of many rural landscapes around the world, promotes high diversification of species and habitats, particularly at small spatial scales [68]. Being located in an underdeveloped area, the low overall intensity of agricultural development may contribute to the high diversification of species and habitats in this river.

However, it should be noted that although the positive impacts of agriculture and aquaculture on fish diversity in the Lixian River was observed, this does not mean that all agricultural and aquaculture activities will yield similar effects. Here, a negative correlation was observed between the aquatic product output and the Chao1 index (Figure 7). The increase in aquatic product output often implies intensified fishing activities and/or fish farming. Overfishing can directly reduce the fish populations, especially for some economically valuable fish species, whose populations struggle to recover and stabilize under extensive fishing pressure, thereby reducing overall fish species richness [69]. At the same time, fishing may lead to the loss of diversity within the ecosystem, with particularly greater impacts observed when fishing targets are some specific species rather than random ones [70]. Under intensified fish farming, abuse of antibiotics and other drugs can lead to serious deleterious ecological problems, including a decline in fish diversity [71]. In pursuit of high yields, just one or a limited number of species will be commonly selected in aquatic farming, which may change the ecological environment of both the aquaculture area and the surrounding natural water bodies [72]. In addition, escaped farmed fish can compete with wild fish for food and habitats, introduce diseases and parasites, and pose threats to the survival and reproduction of wild fish, ultimately reducing their diversity [73,74]. In the present study, tilapia were found to have established a stable population and become the dominant species in the Lixian River (Figure 2). Therefore, although the appropriate development of economic levels, especially agriculture-related activities, relates to higher fish diversity in the less economic developed regions, overfishing, improper fish farming, and invasive species are still risks that need attention.

### 4.4. Pathways of the Effects of Economic Development on Fish Diversity in the Lixian River

Based on the PLS-PM analysis in the present study, we can conclude that, except for agriculture, which directly influences fish diversity, all three parameters of socio-economic development affect water quality and climate, interacting with each other (Figure 9). This suggests a fundamental effect of water quality on river fish diversity. Indeed, the pH value and dissolved oxygen were key factors affecting fish diversity in this river (Figure 6). With the development of the rural economy, decreased levels of forestry, and increased industrial activity, the intensity and type of agricultural practices have been reported as critical factors affecting freshwater quality [75]. Water quality parameters such as total nitrogen (TN), dissolved oxygen (DO), and so on, have been widely recognized as critical factors in shaping the diversity and biomass of both phytoplankton and zooplankton [76,77,78]. As key elements in the aquatic biosystem, phytoplankton function as the primary producers supporting zooplankton populations, and all plankton serve as trophic resources of many fish species [79,80]. Thus, the biomass and diversity of plankton can directly affect fish communities and biodiversity via trophic cascades [78,81,82]. To determine whether the effects of socio-economic development in this river were mediated by the water quality–plankton pathway, analyses of the biomass and diversity of plankton, as well as the factors affecting their distribution were also performed. The results showed that both phytoplankton and zooplankton densities and biomass demonstrated higher values in the upper reaches compared to the lower reaches, and total nitrogen (TN) and dissolved oxygen (DO) represented the primary environmental drivers shaping the plankton community structure in this system (Figure S6). Combined with what we have discussed above, this provides proof that economic activities can systematically alter aquatic community structures in the Lixian River basin through water quality-mediated ecological pathways.

## 5. Conclusions

Based on eDNA technology and multiple statistical analyses, a higher fish diversity was observed in the upper and lower reaches of the Lixian River, which are located in economically underdeveloped areas. Climatic factors are the key factors affecting fish diversity in the Lixian River. However, in this less economically developed river basin, local economic activities, especially the development of agriculture and aquaculture, have an indirect positive effect on fish diversity. Water quality–plankton might be the mediators of both climate and economic activities affecting fish diversity in the Lixian River.

## Data Availability

The original contributions presented in this study are included in the article/supplementary material. Further inquiries can be directed to the corresponding authors.

## Supplementary Materials

The following supporting information can be downloaded at: https://www.mdpi.com/article/10.3390/ani15162416/s1, Figure S1: Dilution curve and species accumulation curve of the tested eDNA samples in the Lixian River; Figure S2: Box (A) and line plot (B) Per capita GDP of Lixian River basin and Yunnan province in recent 10 years; Figure S3: Heat map of the correlation between economic indicators in Li Xian River; Figure S4: Generalized linear model analysis of fish diversity indices and indicators of economic activities in the group with lower maximum temperature (results of non-significant parts); Figure S5: Generalized linear model analysis of fish diversity indices and indicators of economic activities in the group with higher maximum temperature (results of non-significant parts); Figure S6: Phytoplankton and zooplankton in the Lixian River and their driving environmental factors; Table S1: Indicators of water quality in the Lixian River; Table S2: Data of economic indicators in the Lixian River used in the present study; Table S3: Table of HTMT (Heterotrait-Monotrait Ratio) Values in the PLS-SEM Model.

## Abbreviations

The following abbreviations are used in this manuscript:

eDNA	Environmental deoxyribonucleic acid
OTUs	Operational taxonomic units
Temp	Water temperature
Tmmax	Maximum temperature
Tmmin	Minimum temperature
DO	Dissolved oxygen
SAL	Salinity
EC	Electrical conductivity
TN	Total nitrogen
PERMANOVA	Permutational multivariate analysis of variance
PCR	Polymerase chain reaction
ASVs	Amplicon sequence variants
PCoA	Principal Co-ordinates Analysis
CCA	Canonical Correlation Analysis
PLS-PM	Partial least-squares path modeling
