# Supplementary material for: Positive Correlation Between Economic Activities and Fish Diversity in Small River Basins of Less Developed Regions: A Case Study of the Lixian River Basin
Source: Animals (Basel). 2025 Aug 18;15(16):2416. doi: 10.3390/ani15162416 (PMC12382944; doi:10.3390/ani15162416)
Supplement: Supplementary file 1 [file animals-15-02416-s001.zip › animals-3783174-supplementary.pdf]

Figure S1 Dilution curve and species accumulation curve of the tested eDNA samples in the Lixian River

A: Dilution curve of samples collected in summer; B: Dilution Curve of samples collected in winter; C: Species accumulation curve samples collected in summer; D. Species accumulation curve samples collected in winter.

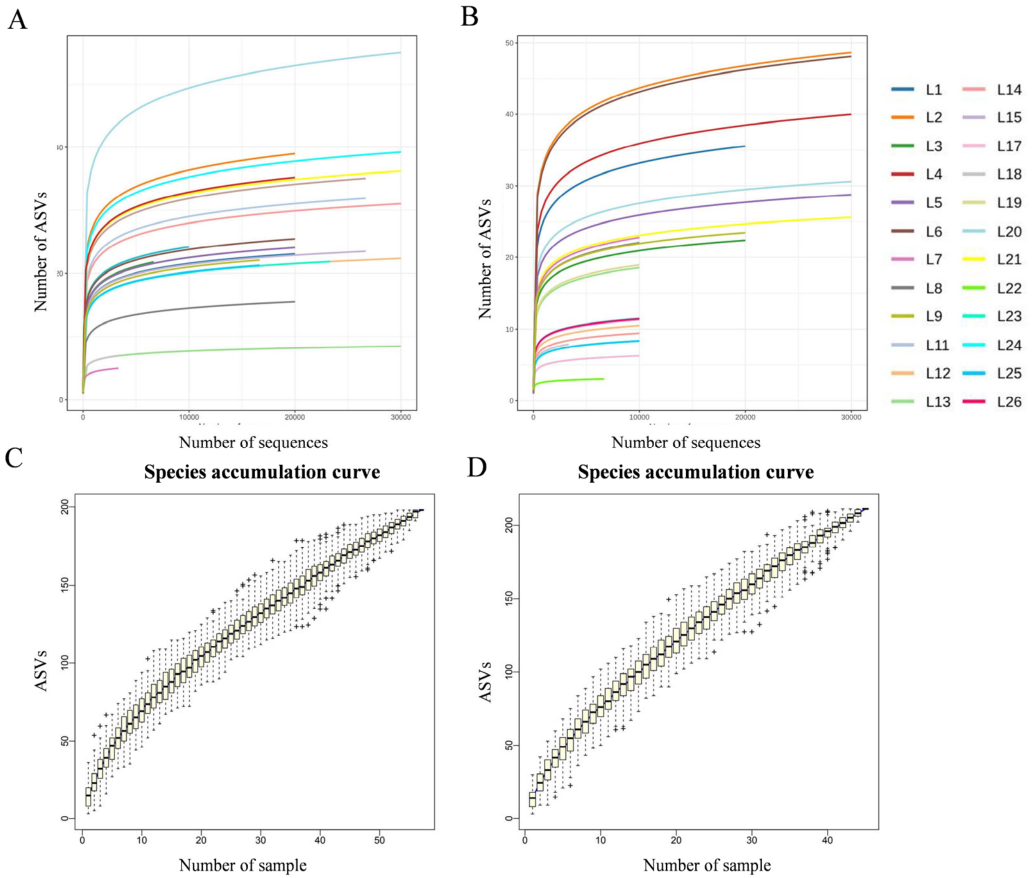

Figure S2 Box (A) and line plot (B) Per capita GDP of Lixian River basin and Yunnan province in recent 10 years. One-way ANOVA was used for the statistics, *: P<0.05; * *:P<0.01.

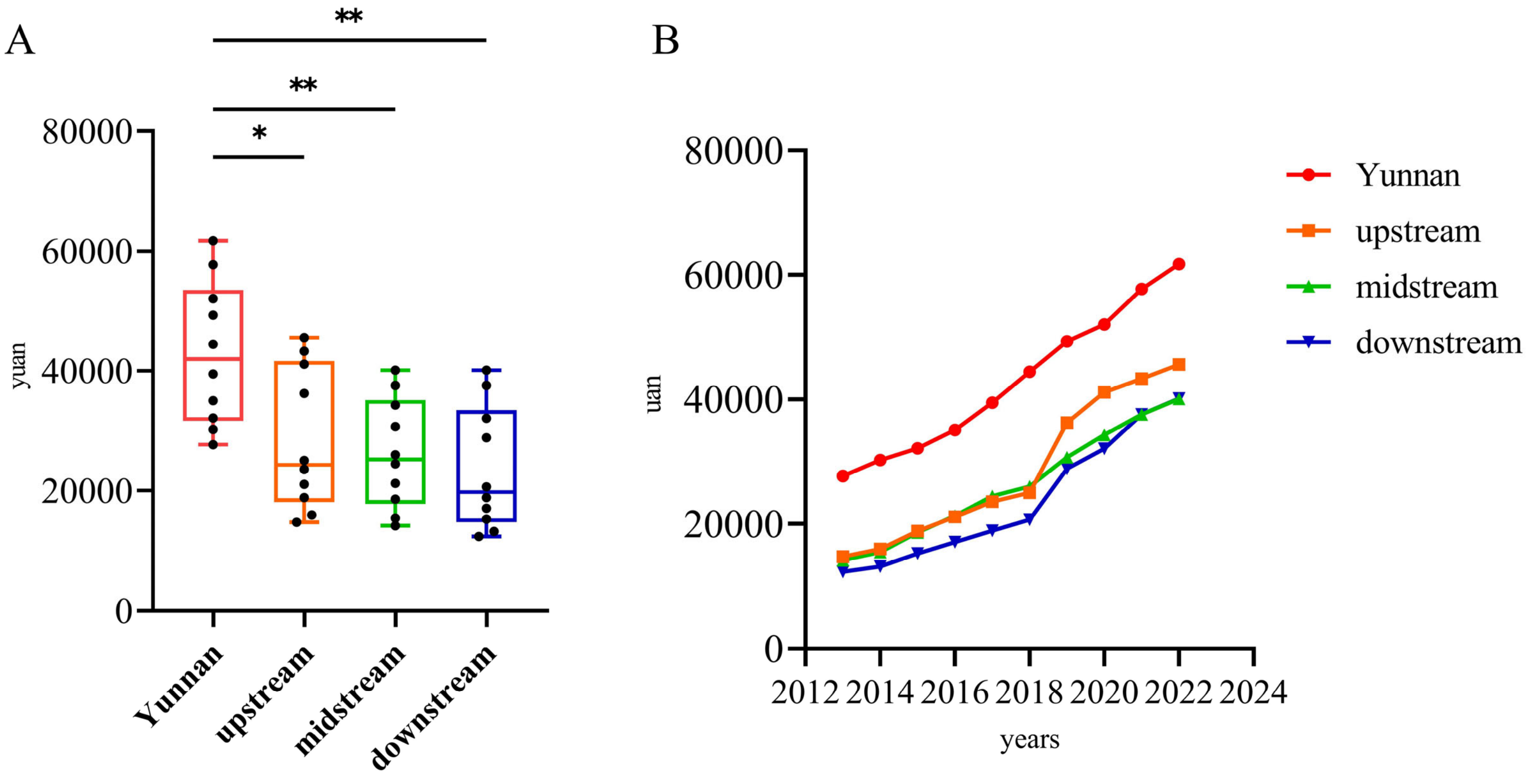

Figure S3 Heat map of the correlation between economic indicators in Li Xian River.

Pearson analysis were applied; the red to blue represent the correlation coefficients from high to low, and the numbers in the box represent the correlation coefficients.

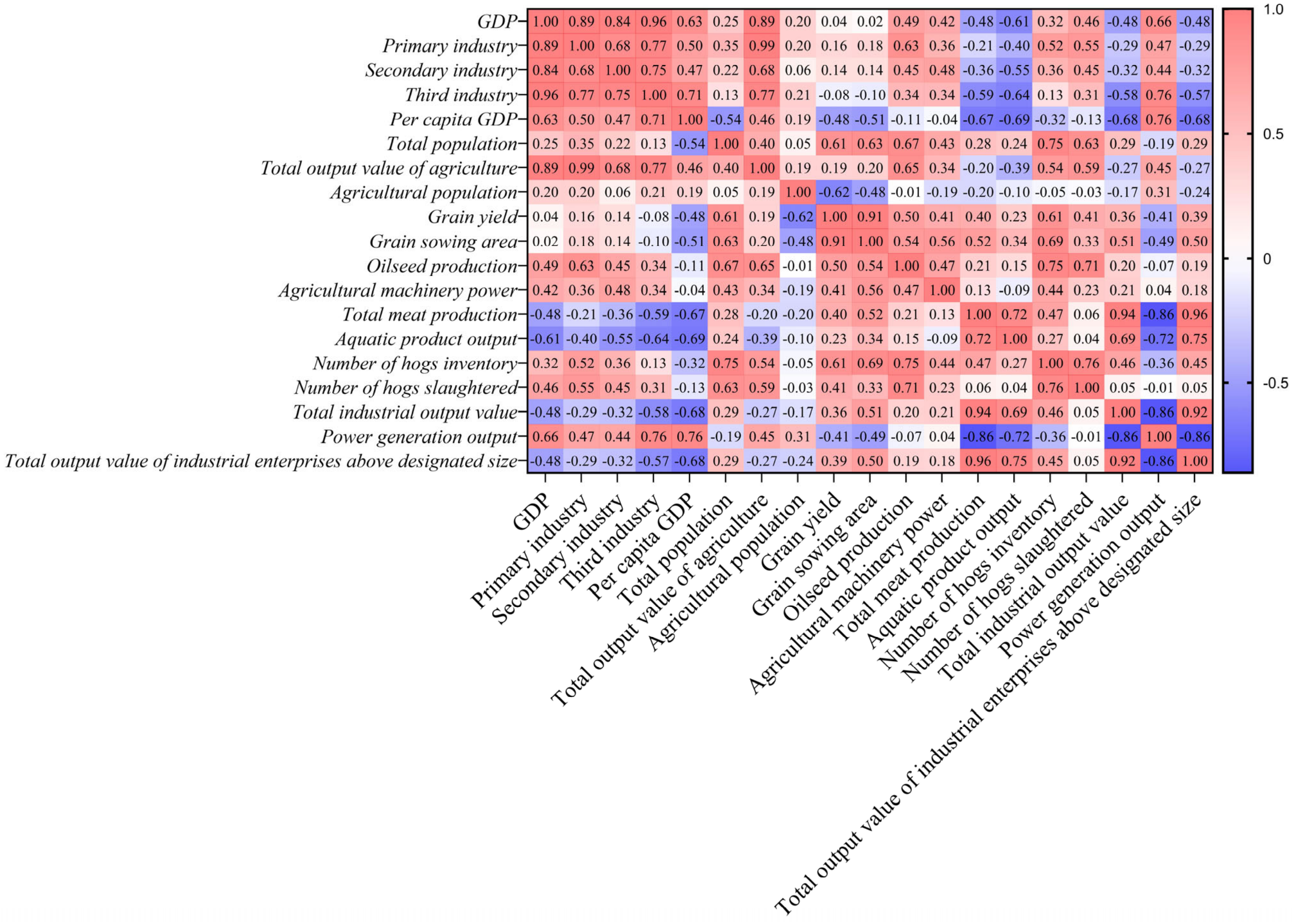

Figure S4 Generalized linear model analysis of fish diversity indices and indicators of economic activities in the group with lower maximum temperature (results of non-significant parts). A-B:GDP-Simpson, Pielou; C-F:Total population-Simpson, Pielou,Chao1, Shanon; G-I: Agricultural population-Pielou, Chao1,Shannon; J-K: Grain yield-Pielou, Chao1; L: Hogs slaughtered-Pielou; M-O: Output of aquatic product -Simpson, Pielou, Shannon ; P-R: Industrial output–Simpson, Chao1, Shannon; S: Industrial output–Pielou.

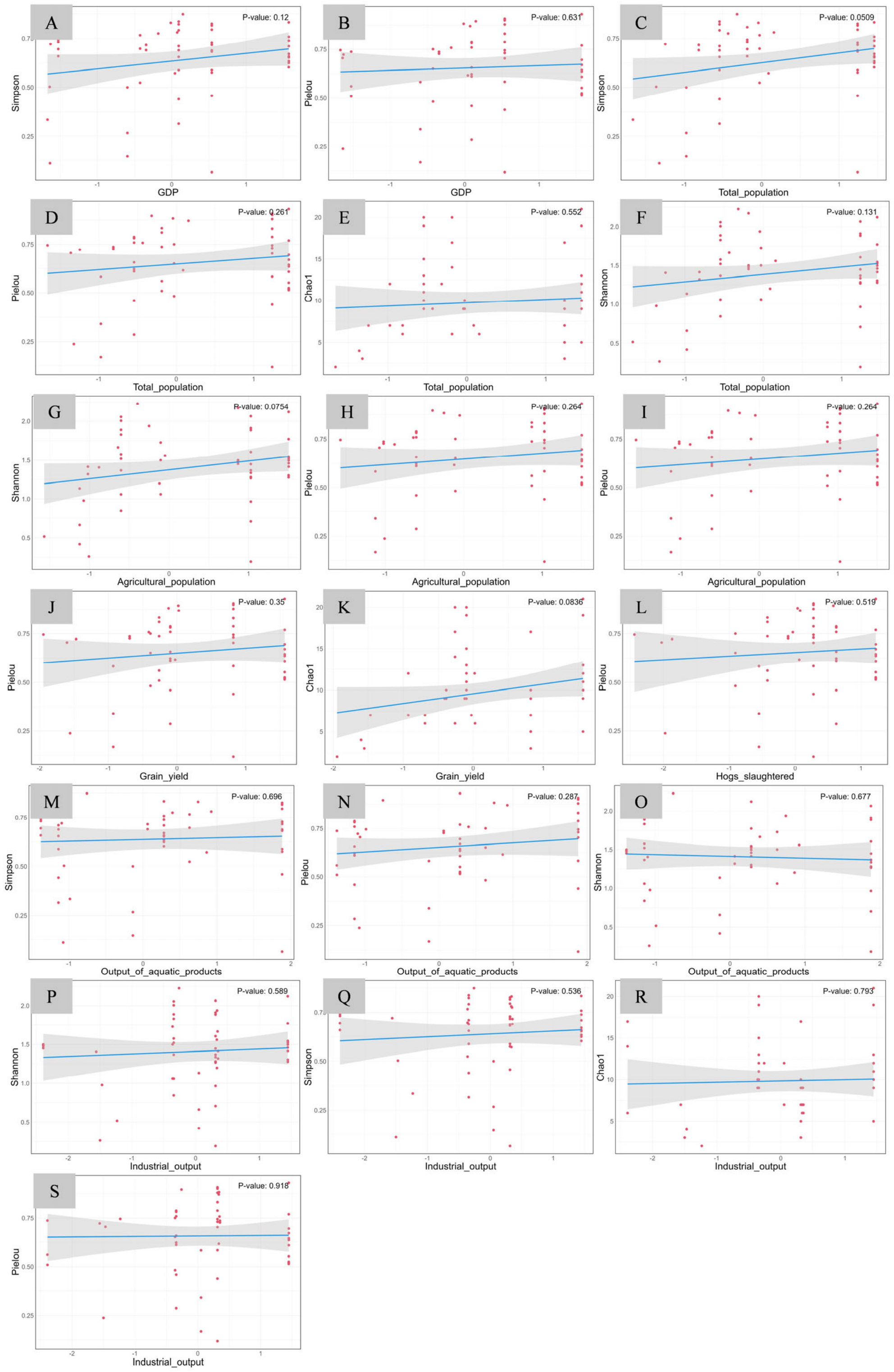

Figure S5 Generalized linear model analysis of fish diversity indices and indicators of economic activities in the group with higher maximum temperature (results of non-significant parts). A:GDP-Pielou; B: Total population-Pielou; C: Agricultural population-Pielou; D: Grain yield-Pielou; E-H: Hogs slaughtered-Chao1, Shannon, Simpson, Pielou; I-L: Output of aquatic product -Chao1, Shannon, Simpson, Pielou; M: Industrial output–Chao1.

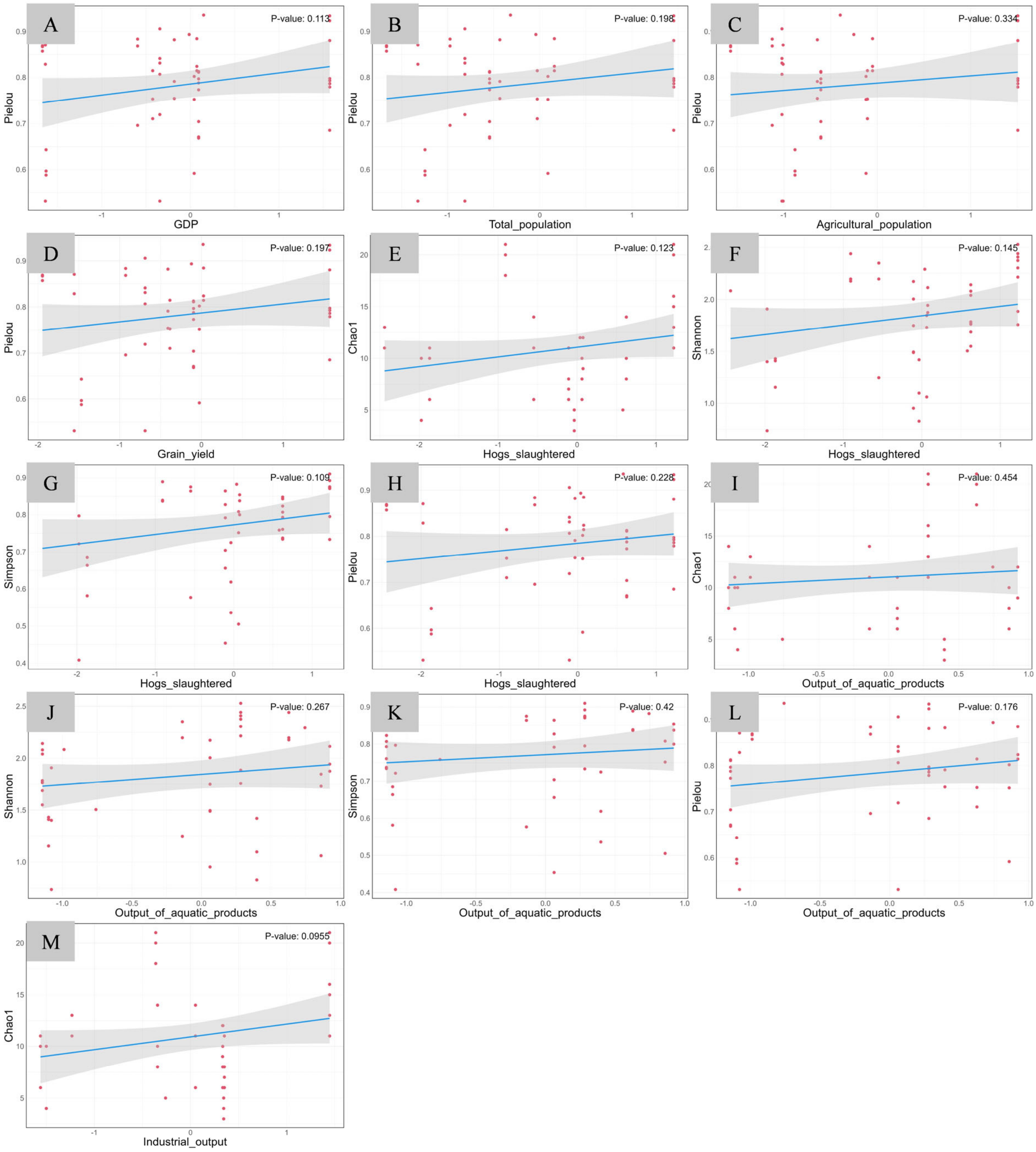

Figure S6 Phytoplankton and zooplankton in the Lixian River and their driving environmental factors.

A: density of phytoplankton; B: biomass of phytoplankton; C: density of zooplankton; D: biomass of zooplankton; E: Redundancy analysis (RDA) based on phytoplankton density; F: RDA based on phytoplankton biomass; G: RDA based on zooplankton density; H: Redundancy analysis RDA based on zooplankton biomass.

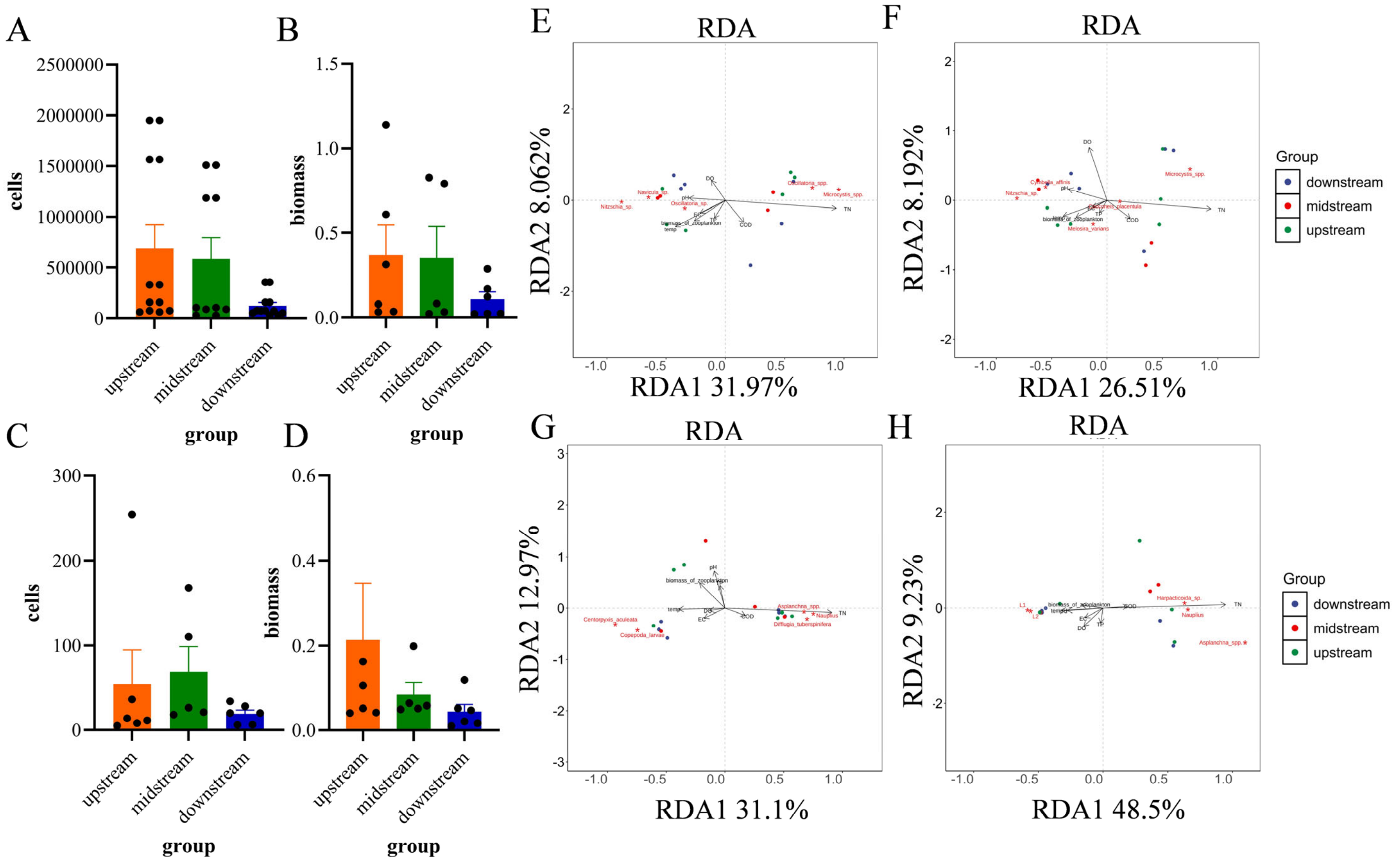

Table S1 Indicators of water quality in the Lixian River

Season	Index	Maximum value	Minimum value	Average value	Variance
Summer	Temp	29.00	20.20	26.20	5.17
	Do	10.34	6.59	7.54	0.57
	EC	0.19	0.06	0.11	0.00
	SAL	482.00	173.00	305.50	3345.16
	pH	9.35	8.54	8.88	0.05
Winter	Temp	28.30	18.60	22.50	6.55
	Do	11.30	6.72	8.55	0.75
	EC	376.00	163.00	263.73	2162.29
	SAL	173.00	74.00	117.95	490.23
	pH	7.99	7.28	7.86	0.02

Note: Temp represents water temperature (ºC), Do represents dissolved oxygen (mg/L), EC represents electrical conductivity (uS), SAL represents salinity (ppt), and pH represents acidity and alkalinity.

Table S2 Data of economic indicators in the Lixian River used in the present study

Group	Index	Maximum value	Minimum value	Average value	Variance
Overall economy	GDP	122.34	20.69	56.96	24.36
	Primary industry	39.74	6.57	17.32	8.17
	Secondary industry	22.75	8.33	15.83	3.97
	Third industry	64.41	5.10	23.80	14.31
	Per capita GDP	58297.00	9134.00	26436.90	12275.56
	Total population	36.59	10.50	22.02	7.59
Agriculture	Total output value of agriculture	66.17	13.00	29.31	13.06
	Agricultural population	33.66	5.87	16.36	7.81
	Grain yield	19.18	3.90	11.39	4.44
	Grain sowing area	4.65	1.36	3.23	1.09
	Oilseed production	4405.00	279.00	2290.95	1079.40
	Agricultural machinery power	35.00	5.00	23.00	8.93
Breeding industry	Total meat production	35694.00	6301.00	23921.92	8707.90
	Aquatic product output	3.30	0.25	1.17	0.82
	Number of hogs inventory	45.99	4.66	25.51	12.54
	Number of hogs slaughtered	44.55	5.51	24.97	11.27
Industry	Total industrial output value	37.49	5.87	15.62	5.73
	Power generation output	34.44	0.03	8.83	10.83
	Total output value of industrial enterprises above designated size	230286.00	58709.00	151179.60	42823.26

Table S3 Table of HTMT (Heterotrait-Monotrait Ratio) Values in the PLS-PM Model

group	Water quality	Agriculture	Climate	Diversity	Overall economy	Industry
**Water quality**						
**Agriculture**	0.479					
**Climate**	0.865	0.186				
**Diversity**	0.741	0.221	0.397			
**Overall economy**	0.237	0.668	0.103	0.101		
**Industry**	0.365	0.705	0.1	0.153	0.716	

Plankton Sampling Methods

Sampling sites

Plankton sampling campaigns were conducted at eight designated monitoring stations (L2, L5, L6, L11, L19, L23, L25, and L26) during distinct hydrological seasons in May and October 2024.

Sample collection

Surface water samples (0.5 m depth) were collected using a 2-liter horizontal water sampler. For phytoplankton collection, a conical plankton net (mesh aperture: 64 μm) was deployed for passive filtration. The concentrated phytoplankton biomass retained in the cod-end was quantitatively transferred to 50 mL amber glass vials and immediately preserved with neutral buffered 10% formaldehyde (final concentration: 4% v/v). Zooplankton sampling employed an analogous protocol utilizing a larger mesh size (112 μm) to optimize retention efficiency for metazoan organisms.

Analysis

Taxonomic identification and quantitative analysis were performed using a CX33 Olympus biological microscope equipped. Plankton density (cells L−1) was calculated using the formula:

$$D = \frac{N \times C}{V}$$

where D = density, N = enumerated cells, C = concentration factor, and V = filtered water volume.

Biomass Quantification

The biomass of Plankton is calculated from the cell volume of Plankton, which is determined by the average cell dimensions for each species Plankton cell dimensions was determined through geometric modeling based on morphological approximations to standard geometric shapes (e.g., spheres, cylinders, ellipsoids). Critical dimensions (length, width, diameter) were measured using ImageJ v1.53 software (NIH) following digital capture at specific magnification.
